# Association of Toll-like receptors 4 (TLR-4) gene expression and polymorphisms in patients with severe asthma

**DOI:** 10.25122/jml-2021-0173

**Published:** 2021

**Authors:** Wasan Shukur, Kifah Alyaqubi, Rasha Dosh, Ali Al-Ameri, Hayder Al-Aubaidy, Rehab Al-Maliki, Ali Aridhee, Rawaa Al-Fatlawi, Najah Hadi

**Affiliations:** 1. Department of Medical Microbiology, University of Kufa, Kufa, Iraq; 2. Department of Middle Euphrates, Cancer Research Unit, University of Kufa, Kufa, Iraq; 3. Department of Anatomy and Histology, Faculty of Medicine, University of Kufa, Kufa, Iraq; 4. Department of Medicine, La Trobe University, Melbourne, Victoria, Australia; 5. MD Anderson Cancer Center, Orlando, Florida, United States of America; 6. Department of Pharmacy, The Islamic University, Najaf, Iraq; 7. Department of Medicine, Al-Nahrain University, Baghdad, Iraq; 8. Department of Clinical Laboratory Sciences, Faculty of Pharmacy, University of Kufa, Kufa, Iraq; 9. Department of Pharmacology and Therapeutics, University of Kufa, Kufa, Iraq

**Keywords:** asthma, polymorphism, TLR-4 asthma, Toll-like receptors, innate immunity, IgE

## Abstract

Innate immunity plays a central role in the pathogenesis of severe asthma, and it is closely linked to elevated IgE and Toll-like receptor 4 (TLR-4) levels. However, there is a scarcity of information about the association of the TLR-4 receptor polymorphism in the pathogenesis of severe asthma. This study highlights the level of gene expression of different alleles in asthmatic patients compared to healthy control individuals. This was a randomized control trial, which included 150 patients with asthma (with high serum levels of IgE) with a matching 150 healthy control individuals. Participants had a series of blood tests to measure various immune parameters: interleukin-1 (IL-1), interleukin-6 (IL-6), tumor necrosis factor (TNF), intercellular adhesion molecule-1 (ICAM1) and detect allele type and gene expression of the TLR-4 gene. Patients with asthma had significantly higher levels of IL-8 when compared to the healthy control participants. In addition, in the rs91 genotyping, there were significant differences in the levels of IL-8 and TNF between CC and TT genotyping. While in rs90 TLR-4, TNF levels were significantly higher in AA vs. AG and GG genotypes among the asthmatic patients when compared to the control group. The results showed that in TLR-4, rs4986791 were significantly associated with asthma risk. Polymorphisms in TLRs play essential roles in asthma.

## Introduction

Bronchitis and reverse airway obstruction are among the most important features of asthma, and it has significantly increased in recent decades [1]. Asthma has become a significant health problem, increasing health expenditures and affecting the quality of life and work [2]. The link between asthma and innate immunity has been previously shown by many studies, with evidence that the response to antivirals decreases bronchial cells in asthma patients, as well as increases the expression of receptors in severe asthma. This indicates that innate immunity has active participation in the exacerbation of asthma, and innate immunity represents the first line of defense against microbes. The Toll-like receptor (TLR) family comprises receptors belonging to innate immunity with the mission of identifying microbes and activating immunity with both fungal and adaptive types by different cells in the airway, including fat cells, macrophages, and epithelial cells. In addition to smooth muscles, Toll-like receptor-4 (TLR-4) recognizes lipopolysaccharides (LPS) [3, 4]. Genetic and environmental interferences are vital in the severity and development of asthma. Activation of innate immunity, especially TLR-4 receptors, is dependent on air pollutants, such as ozone, CO_2_, and N2.

TLR-4 protects against Gram-negative bacteria by possessing LPS receptors. Also, the molybdenum produced from compounds can interfere with LPS that activate TLR-4 and cause acute or chronic inflammation, depending on the type of single nucleotide polymorphisms (SNPs) for TLR-4 [5, 6]. Modern medicine actively seeks to predict the interventions and the possibility of treatments through the knowledge of individual genetic features and disease incidence. Despite this, few researchers are interested in the interactions between the environment and personal genes in complex diseases such as asthma. Accordingly, we expect that the level of TLR-4 will be increased in the airways of asthmatic patients with severe exacerbation of the condition. Therefore, we aimed to compare the polymorphisms and expression of TLR-4 in asthma patients with healthy controls. TLRs are pivotal innate immune receptors that induce inflammatory responses, and their expression and activation are increased in disorders with an excess of inflammation, including asthma and its complications [3, 7]. Genetic polymorphisms are variations within the genome that can affect the innate immunity pathway and the extent of its activation and response upon exposure to stimuli and, consequently, influence the lifetime risk of a given disease [8]. The difference in the family of innate immune receptors can be partly explained as representing the inherited difference in predisposition to developing diseases such as infections or different immune-self-diseases [5]. Of the common nucleotides of the TLR-4 gene, we identified two positions in the exon3 expression region, and this mutation switches the amino acid at position 299 (Asp299Gly) and position 399 (Thr399lle). It has been shown that the ratio of the extracellular domain changes by changing these alleles, Gly 299 and lle399, which affects the binding and hence, the resultant immune response [9–11].

## Material and Methods

This is a randomized control trial that included 150 patients with severe asthma who were invited to participate in this study at the Al-Sadr Teaching Hospital, Al-Najaf, Iraq. A matching group of 150 healthy control participants was also included in this study. Power calculation was performed using the equation described by Menashe *et al.* in their study [12].

Five milliliters (ml) of venous blood were collected from patients by venipuncture. A volume of 5 ml of blood was drawn. Then, blood was divided into 2 aliquots, one sample of 1 ml and other of 4 ml. Sample A was added to EDTA tubes for DNA isolation used in RFLP-PCR or SSP-PCR genotyping studies Sample B was added to EDTA tubes for RNA isolation used for RT-PCR. Plasma was separated by centrifugation at 2500 rpm for 10 minutes and divided into small (200 μl) aliquots that were kept at -20 C° to be used for different investigations.

We excluded form our study patients that had diabetes mellitus (DM), heart failure, inflammatory disorders, abnormal liver ond/or thyroid function, malabsorption. Also, those receiving steroid therapy, anti-inflammatory, antihypertensive, or hypolipidemic drugs were excluded. Pregnant women, smokers and/or alcohol drinkers were not considered.

### Isolation of total RNA and synthesis of cDNA

Total RNA was isolated using Guy’s protocol for total RNA [13]. The total RNA was reversely transcribed to complementary DNA (cDNA) using the WizScript™ RT FDmix Kit. According to the manufacturer’s instructions, the procedure was carried out in a reaction volume of 20 μl [13].

### Quantitative real-time polymerase chain reaction (qPCR)

The expression levels of the TLR-4 gene were estimated using qRT-PCR, and SYBR Green assay was used to record the expression of the gene of interest. The computer program Primer Express was used to design the Primers and probes (USCS and 3plus, USA). Duplicate reactions showing differences of more than 0.3Ct were repeated. Two non-template controls were also included in each run. The mRNA levels of endogenous control gene were amplified and normalized the mRNA levels of the TLR-4 gene and corrected the synthesis of cDNA as well as the calculations descriptions. Two selected SNPs, (Asp299Gly) TLR4 (+986 A/G) exon 3, and (Thr399Il3) TLR4 (+1196 C/T) exon 3, were commonly associated with altered levels of TLR-4 when using real-time PCR allelic discrimination. During PCR, if the target of interest is present, the probe specifically anneals to the target; if the probe hybridizes to the target (5’ to 3’ nucleolytic activity of the AmpliTaq Gold), the polymerase enzyme cleaves the probe between the reporter and the quencher only.

### Statistical Analysis

Statistical analyzes were done using the Statistical Package for the Social Sciences (SPSS) software. An intergroup chi-square analysis was performed for genotype comparison and allele repeatability and to verify the association with the clinical parameters. An analysis of variance was used. A p-value <0.05 was considered statistically significant.

## Results

The mean values for the rs4986791 (+1196C/T) and rs4986790 (896A/G) variants in the two studied groups are shown in Tables 1 and 2, respectively. Table 3 shows the results of the inflammatory markers used in this study in reference to the rs91 genotype of TLR-4. There was a significant increase in the plasma levels of interleukin-8 (IL-8) and tumor necrosis factor (TNF) in the CC genotype, while the CT genotype showed a smaller rise in the IL-8 levels, but still significant (P<0.005).

**Table 1.** Cycle threshold (CT) value distribution of rs4986791 (+1196C/T) variants in the studied groups.

**Genotypes**		**Mean±SD**	
		**Control Group**	**Asthma Group**
CC		20.80±0.98^a^	23.08±0.05^a^
CT		22.60±1.57^a^	20.55±4.63^b^
TT		22.91±1.47^a^	20.59±3.07^b^
**Total**		20.58±2.02^b^	22.32±3.09^a^
**P**	CC vs. CT	NS	NS
	CC vs. CT	NS	NS
	CC vs. CT	NS	NS

**Table 2.** Cycle threshold (CT) value distribution of rs4986791 (+1196C/T) variants in the studied groups.

**Genotypes**		**Mean±SD**	
		**Control Group**	**Asthma Group**
AA		21.9±1.17^a^	21.80±3.03^a^
AG		21.43±2.36 ^a^	22.13±2.31 ^a^
GG		22.11±2.42 ^a^	21.51±2.71 ^a^
**Total**		21.80±2.31 ^a^	22.20±2.69 ^a^
P	AA vs. AG	NS	NS
	AA vs. GG	NS	NS
	AG vs. GG	NS	NS

**Table 3.** IL-8, IL-1, TNF, ICAM1 concentration in the rs91 genotype in the studied groups.

**Parameters**	**Genotypes**	**Mean±SD**	
		**Control Group**	**Asthma Group**
**IL-8**	CC	40.9±11.9	56.0±25.4 *
	CT	43.6±14.4	51.9±18.7 *
	TT	49.7±22.3	53.2±17.1
	CC	4.1±1.5	3.2±1.1
**P-value**	CC vs. TT	S	NS
	CT vs. TT	NS	NS
	CC vs. CT	NS	NS
**IL-1**	CC	3.7±0.8	4.0±0.6
	TT	3.7±1.3	3.3±0.9
	CT	0.8±0.2	1.1±1.0 *
**P-value**	CC vs. TT	NS	NS
	CT vs. TT	NS	NS
	CC vs. CT	NS	NS
**TNF**	CC	1.0±0.3	1.0±0.2 *
	TT	1.0±0.2	1.4±0.1
	CT	50.2±10.3	44.3±10.3
**P-value**	CC vs. TT	S	NS
	CT vs. TT	NS	NS
	CC vs. CT	NS	NS
**ICAM-1**	CT	44.6±6.1	40.0±7.1
	TT	42.1±10.4	40.8±2.6
	CC vs. CT	NS	NS
**P-value**	CC vs. TT	NS	NS
	CT vs. TT	NS	NS

* Significant differences between the control group and the asthma group. S – significant differences between the genotyping alleles; NS – non-significant differences.

Table 4 shows the results of the inflammatory markers used in this study in reference to the rs90 genotype of TLR-4. There was a significant increase in the plasma levels of IL-8, IL-1, and TNF in the GG genotype, while the AA genotype showed only a smaller rise in the IL-1 levels, but still significant (P<0.005).

**Table 4.** IL-8, IL-1, TNF, ICAM1 concentration in the rs91 genotype in the studied groups.

**Parameters**	**Genotypes**	**Mean±SD**	
		**Control Group**	**Asthma Group**
**IL-8**	AA	40.9±11.9	56.0±25.4 *
	AG	43.6±14.4	51.9±18.7 *
	GG	49.7±22.3	53.2±17.1
		
**P-value**	AA vs. GG	NS	NS
	AG vs. GG	NS	NS
	AA vs. AG	NS	NS
IL-1	AG	3.69±1.30	3.52±1.16
	GG	4.66±0.96	2.8±1.22*
	AA	1.0±0.26	2.13±0.2*
**P-value**	AA vs. GG	NS	NS
	AG vs. GG	NS	NS
	AA vs. AG	NS	NS
**TNF**	AG	0.95±0.24	1.1±0.25
	GG	0.96±0.09	1.11±1.91 *
	AA	49.70±8.92	41.59±10.79
**P-value**	AA vs. GG	NS	S
	AG vs. GG	NS	NS
	AA vs. AG	NS	S
**ICAM-1**	AG	46.82±9.55	42.45±7.29
	GG	47.48±9.18	43.73±8.93
	AA vs. AG	NS	NS
**P-value**	AA vs. GG	NS	NS
	AG vs. GG	NS	NS

* Significant differences between the control group and the asthma group. S – significant differences between the genotyping alleles; NS – non-significant differences.

## Discussion

In this study, patients with asthma showed significantly higher levels of IL-8 than the controls. Regarding the CC genotype, IL-8 concentrations were significantly higher in the asthma group (56.0±25.4) than in the control group (40.9±11.9). Regarding the CT genotype, IL-8 concentration was significantly higher in the asthma group (51.9±18.7) than in the control group (43.6±14.4). These results are in agreement with other studies which reported that patients with asthma had higher levels of IL-8 than normal individuals [14, 15]. In severe cases of asthma, the levels of neutrophils increase in the airways, leading to the production of cytokine IL-8 and exacerbate the degree of asthma [16]. The smooth muscle cells (SMC) and macrophage foam cells express IL-8 in the inflammatory regions, suggesting a function of this cytokine along with tumor necrosis factor-a (TNF-α) and interleukin-1 (IL-1), in the progression of severe asthma [17, 18]. The increased levels of neutrophils can be explained by how increased neutrophils and acid cells are in the sputum of severe asthma patients; also, there are differences in cytoskeletal expression compared to moderate asthma patients [19]. The airway tissue produces several bioactive molecules, including IL-8 and TNF-α, collectively called adipokines, which affect inflammatory sensitivity and may have a powerful role in developing asthma. Moreover, the molecules expressed by the human macrophages can change chronic inflammation and lead to respiratory complications [14].The pro-inflammatory cytokines, particularly IL-8, have been demonstrated to prevent sensitivity mediators transport in airways [19, 20]. The pro-inflammatory particles may have a more important role than anti-inflammatory proteins in the increase of severe hypersensitivity. Moreover, increasing data support the hypothesis that a low-grade pro-inflammatory state correlated with raised visceral fat may induce hypersensitivity. It has been shown that acute IL-8 exposure increases vesicle permeability. Furthermore, ICAM-1 has been proven to be a negative acute-phase protein, so its levels may decrease in response to a person’s cytokine status. A weak inverse association between serum ICAM-1 and serum IL-8 was proved [7]. Nowadays, TLRs are usually identified as key regulators of perceptivity to both infectious and non-communicable diseases. Asthma is a chronic airway inflammatory disease that is influenced by the interplay between genetic factors and exposure to environmental allergens, microbes, or microbial products where TLRs play a pivotal role. TLRs are involved in the innate and adaptive immune responses and the pathogenesis of asthma [8]. In this study, we investigated the role of the *TLR4 Asp299Gly* and Thr399Il3, gene polymorphisms and expression in the susceptibility to the severity of asthma in the Iraqi population. To our knowledge, this is the first study showing the association between *TLR4* SNPs and expression with asthma in the Iraqi population [3]. We identified the frequencies of *TLR4 Asp299Gly* genotypes which were not significant, but frequencies of alleles were significantly different among the studied groups (P=0.51, P=0.000) consecutively. This proves a significant association between *TLR4 Asp299Gly* polymorphism and the presence of asthma. These results disagree with other studies, which proved no significant association between the *TLR2 Arg677Trp* polymorphism and the presence of asthma [21–23]. However, the frequencies of *TLR4 Asp299Gly* genotypes and alleles were significantly different among the studied groups (P=0.003, P=0.000, respectively). These results agree with the study of Yang *et al.* which proved that there was a significant difference between genotypes of *TLR4 Asp299Gly* polymorphism among the groups, but frequencies of alleles were not significantly different [1]; however, this does not agree with Teräsjärvi *et al.* [11]. Furthermore, our study showed that *TLR4 Asp299Gly* polymorphisms were all absent. Our results do not agree with the results of Teräsjärvi *et al.* which did not show a statically significant relationship between *TLR4 Asp299Gly* polymorphism and asthma. In another study, the *TLR-4 Asp299Gly* polymorphism was found, which was similar to our results [10]. In fact, several studies support the role of *TLR-4* in the pathophysiology of asthma [16]. A high level of *TLR-4* has been shown in human allergies, which leads to activation by several bacteria products and some endogenous ligands, including heat shock proteins expressed during arterial injury. This, in turn, leads to the activation of the *NF-kβ* pathway, which activates cytokines, chemokines, and adhesion molecules. Furthermore, it has been proven that *TLR-4* can be regulated under the “hygiene theory,” as would occur in atherosclerosis-prone airway branches [4]. Furthermore, our findings show that the frequencies of *TLR-4* Thr399Ile genotypes and alleles were not significantly different among the studied groups (P=0.41, P=0.32, respectively). Many studies have shown that *TLR-4 Thr399Ile* polymorphism is associated with reduced susceptibility to allergy, while another report has revealed no association between this polymorphism and asthma patients. In a Russian study, the investigation did not demonstrate a significant association of the *TLR4 Thr399Ile* polymorphism with the risk of asthma, and this agrees with our results [23]. *TLR4* is an essential factor in regulating innate immune responses [9]. The Thr399Ile SNPs in the *TLR-4* gene have been reported to be associated with many diseases. The mutant alleles of *TLR-4* SNPs seem to have originated in Africa in an environment where malaria appears as a major evolutionary pressure. The role of Thr399Ile polymorphisms in the pathogenesis of type 2 diabetes remains controversial. Some studies have proven that Thr399Ile polymorphisms are correlated with susceptibility to types of allergy [5]. Interestingly, the high levels of *TLR-4* lead to an increase in asthmatic subjects. The increasing levels of many cytokines such as IL-8, IL-17 and others indicate that hypersensitivity is the probable reason for the activation of TLR4- [9].

## Conclusion

In patients with severe asthma, hypersensitivity is always accompanied by elevated levels of innate cytokines in the blood, which stimulates the expression of *TLR-4* in human monocytes. Activated *TLR-4* mediates the inflammatory response (as manifested by an increased level of IL-8, IL-1, as well as TNF). This response is associated with TLR-4 gene polymorphisms.

## Acknowledgments

### Ethical approval

The approval for this study was obtained from the Human Research Ethics Committee of the University of Kufa (approval ID: MEC-20

### Consent to participate

The participants entered the study voluntarily and their confidentiality was kept.

### Conflict of interest

The authors declare that there is no conflict of interest.
